# Current Tobacco Use Status Associated with Insufficient Sleep Duration and Sleep Problems Among Non-Hispanic Black and Hispanic Men with Chronic Conditions

**DOI:** 10.3390/epidemiologia7050127

**Published:** 2026-09-11

**Authors:** Ashley L. Merianos, Jodi L. Kaminski, Jahmai Irehovbude, E. Melinda Mahabee-Gittens, Ledric D. Sherman, Caroline D. Bergeron, Matthew Lee Smith

**Affiliations:** 1 School of Human Services, University of Cincinnati, Cincinnati, OH 45221, USA; 2 Division of Emergency Medicine, Cincinnati Children’s Hospital Medical Center, University of Cincinnati College of Medicine, Cincinnati, OH 45229, USA; 3 Department of Health Behavior, School of Public Health, Texas A&M University, College Station, TX 77843, USA; 4 LIFE Research Institute, University of Ottawa, Ottawa, ON K1N 6N5, Canada; 5 School of Public Health—Bloomington, Indiana University, Bloomington, IN 47405, USA

**Keywords:** ethnic and racial minorities, adult, sleep duration, sleep quality, tobacco use, surveys and questionnaires

## Abstract

*Background and Objectives:* This study aimed to examine the associations between exclusive cigarette smoking, dual-tobacco use, and poly-tobacco use and sleep duration and sleep problems among non-Hispanic Black and Hispanic men with chronic conditions. *Materials and Methods:* Cross-sectional, internet-administered survey data were collected in September–November 2019 from 1673 U.S. non-Hispanic Black and Hispanic men aged ≥40 years. Current (past 30-day) tobacco use was classified as non-tobacco use, exclusive combustible cigarette smoking, dual-tobacco use (cigarettes plus one additional tobacco product), and poly-tobacco use (cigarettes plus ≥ 2 additional tobacco products). Other tobacco products included cigars, electronic cigarettes, hookah, pipes, and bidis. Insufficient sleep duration was classified as <7 h, and sleep problems were defined as having sleep concerns in the past week. Two multivariable logistic regression models were performed to estimate adjusted odds ratios (AORs) and 95% confidence intervals (95%CIs) for insufficient sleep and sleep problems while adjusting for sociodemographics, body mass index (BMI), and number of chronic conditions. Participants that were missing data on tobacco use, sleep outcomes, and BMI were excluded. *Results:* Overall, 46.9% of participants reported insufficient sleep duration, and 46.2% reported sleep problems. Exclusive combustible cigarette smokers (AOR = 1.43, 95%CI = 1.07–1.92) and dual-tobacco users (AOR = 1.56, 95%CI = 1.06–2.28) had significantly higher odds of insufficient sleep than non-tobacco users. Dual-tobacco users (AOR = 1.96, 95%CI = 1.32–2.91) and poly-tobacco users (AOR = 1.66, 95%CI = 1.01–2.74) had significantly higher odds of sleep problems than non-tobacco users. *Conclusions:* Findings suggest that cigarette smoking, whether exclusively or in combination with other tobacco products, may exacerbate sleep disparities among middle-aged and older non-Hispanic Black and Hispanic men with chronic conditions.

## 1. Introduction

Adequate sleep is essential for maintaining physical and emotional health [1,2]. Sleep quantity refers to the total duration of sleep within a 24 h period, and sleep quality encompasses shorter sleep latency, fewer nighttime awakenings, and reduced wakefulness after sleep onset [3]. Both the quantity and quality of sleep contribute significantly to overall well-being [4]. The National Sleep Foundation recommends that adults aged 18–64 years obtain 7–9 h of sleep per day [5]. Despite these recommendations, approximately one in three adults report insufficient sleep duration [6]. The prevalence of insufficient sleep is higher among men (37%) and adults aged 45–64 years (39%) [7]. Sleep quality problems are also common in the United States (U.S.), with 14.5% of adults reporting difficulty falling asleep and 17.8% reporting trouble staying asleep [8]. These issues vary by sex and race and/or ethnicity [8]; for example, non-Hispanic Black adults are more likely to report difficulty staying asleep compared to Hispanic adults [8]. Poor sleep quantity and quality are associated with short- and long-term adverse health outcomes, including impaired cognitive function, weakened immune response, and increased risks of diabetes, metabolic disorders, depression, hypertension, and cardiovascular disease [9].

Despite sustained declines in tobacco use among adults over the last few decades, tobacco continues to be the leading cause of preventable morbidity and mortality in the U.S. [10]. In 2023, approximately one in five adults (19.5%) reported current tobacco use of any type; combustible cigarettes were the most used tobacco product (7.9%), followed by electronic cigarettes (e-cigarettes; 4.1%) [11]. Additionally, about 2% of adults reported dual use of two or more tobacco products [11]. Importantly, one in four U.S. adults with at least one chronic disease reported smoking, and this prevalence has remained unchanged despite the overall decline in smoking rates [12]. Data from the 2021 National Health Interview Survey indicate that current tobacco use was most prevalent among men (24.1%) and varied by race and/or ethnicity, with a higher prevalence reported among non-Hispanic Black adults (18.1%) than among Hispanic adults (12.4%) [13]. Among adults who use tobacco, 18.1% reported dual use of two or more products, and among these individuals, 14% reported poly use of three or more tobacco products [13]. These patterns highlight the need to consider tobacco use behaviors when examining health-related outcomes, such as sleep quantity and quality, particularly among men and those living with chronic conditions.

Evolving evidence suggests that tobacco use is a risk factor for sleep problems [14,15]; however, the mechanisms are not completely understood. Nicotine may impair sleep quality in smokers through multiple pathways, including its effect on circadian rhythms, nighttime cravings, and withdrawal [16]. Studies suggest that the relationship between nicotine and sleep quality can be bidirectional, meaning that nicotine leads to sleep problems and sleep problems lead to increased smoking/nicotine use [17,18]. Thus, combustible cigarette smoking has been associated with poor sleep quantity and quality among U.S. adults, but emerging products (e.g., e-cigarettes) and multi-product use patterns may differentially affect sleep, especially among racially and ethnically diverse men with chronic conditions. However, much of the literature has focused on exclusive combustible cigarette smoking, with less attention paid to dual-tobacco and poly-tobacco use patterns and their associations with sleep quantity and sleep problems. This is important because racially and ethnically diverse men with chronic conditions have a high burden of tobacco use and sleep disturbances [8,12,13]. Addressing this gap in the literature on the relationship between dual-tobacco and poly-tobacco use patterns and sleep duration and sleep problems among racially and ethnically diverse men with chronic health conditions is important for informing tobacco prevention and cessation strategies and for improving sleep health among this population, which is at an elevated risk. Therefore, the objective of this study was to assess the associations between exclusive cigarette smoking, dual-tobacco use, and poly-tobacco use and sleep duration and sleep problems among non-Hispanic Black and Hispanic men with one or more chronic conditions.

## 2. Materials and Methods

### 2.1. Participants and Procedures

A cross-sectional, internet-administered survey was completed by non-Hispanic Black and Hispanic men aged ≥40 years with at least one chronic condition residing in the United States from September 2019 to November 2019. The survey instrument was developed by the study team; detailed descriptions of survey development and study procedures have been published previously [19]. Briefly, the parent study used previously validated measures to examine health behaviors such as tobacco and sleep, health status, and multi-level facilitators and barriers to chronic disease management [19]. Although the parent study’s questionnaire was not independently validated as a single instrument, it incorporated questions from established measures. Participants were recruited using a Qualtrics online research panel, a successful methodology used to efficiently reach demographically targeted populations, and required about 30 min of participants’ time to complete [20].

**Figure 1:**
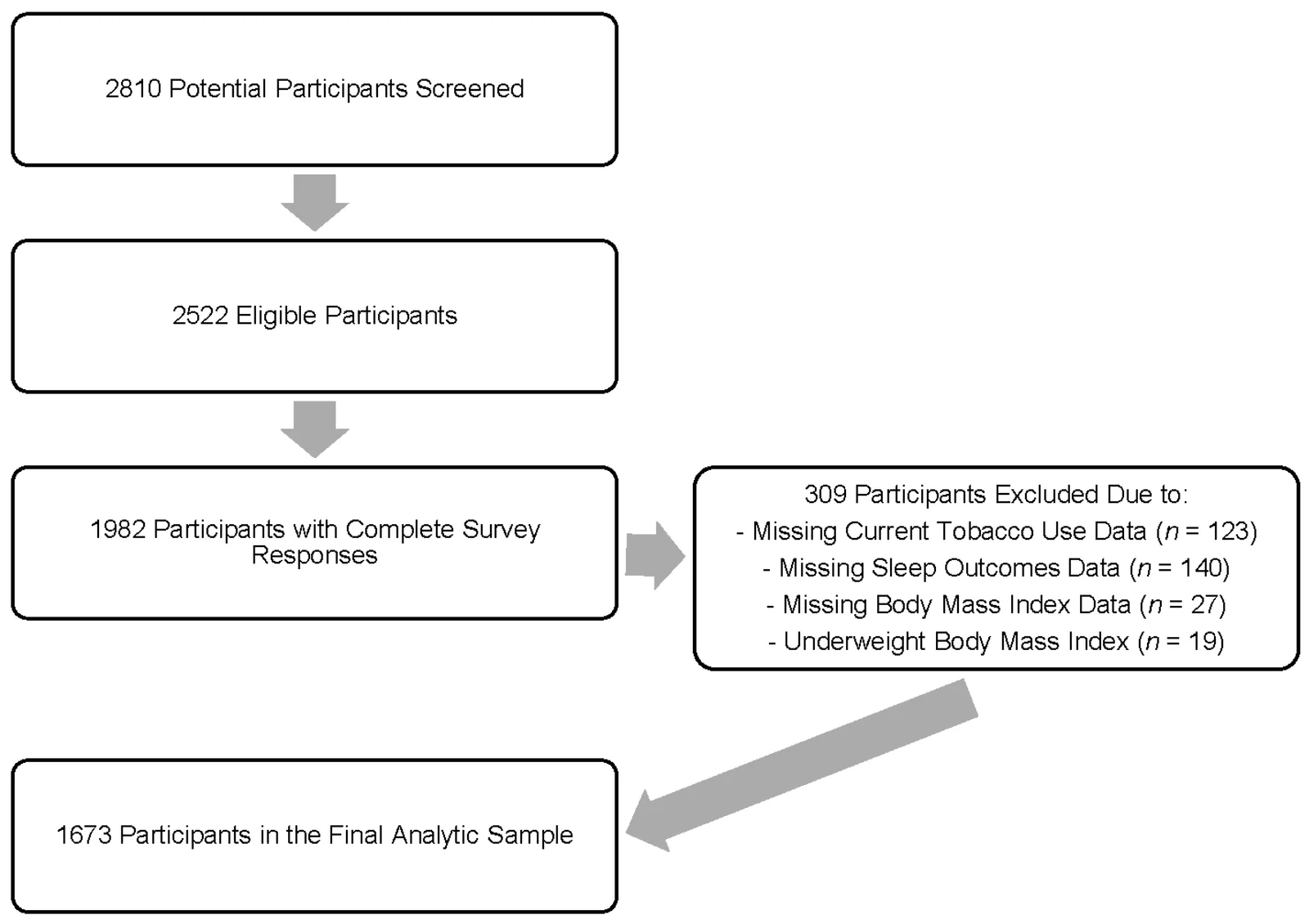
Study participant flowchart illustrating participant screening, eligibility, exclusions, and final analytic sample.

Of the 2810 potential participants screened, 2522 (89.8%) met the eligibility criteria of the parent study, which included self-identifying as non-Hispanic Black or Hispanic, being 40 years of age or older, having at least one chronic condition, and the ability to read English (Figure 1). Following the quality assurance procedures conducted by Qualtrics, 1982 participants (78.6%) provided complete data. The final analytic sample for this secondary study included 1673 participants after excluding those with missing data on current tobacco use (*n* = 123), sleep measures (*n* = 140), and body mass index (BMI, *n* = 27). We also excluded those who were underweight (BMI < 18.5 kg/m^2^) because of the small sample size (*n* = 19).

### 2.2. Measures

#### 2.2.1. Current Tobacco Use Patterns

Participants reported current tobacco use, defined as past 30-day use, in response to questions adapted from the Centers for Disease Control and Prevention’s (CDC’s) Behavioral Risk Factor Surveillance System (BRFSS) [21]. Specifically, participants were asked whether they had used the following combustible and electronic tobacco products during the past 30 days: cigarettes; cigars, cigarillos, or little cigars; e-cigarettes; hookah or waterpipe tobacco; pipes filled with tobacco (excluding waterpipes); and bidis.

Based on their responses, participants were classified into four mutually exclusive categories based on any self-reported current tobacco use during the past 30 days: (1) non-tobacco use, defined as no reported use of any tobacco product; (2) exclusive cigarette smoking, defined as current use of combustible cigarettes only; (3) dual-tobacco use, defined as current use of combustible cigarettes plus one additional tobacco product; and (4) poly-tobacco use, defined as current use of combustible cigarettes plus two or more additional tobacco products. Additional tobacco products for dual-tobacco and poly-tobacco use categories included cigar products, e-cigarettes, hookah or waterpipe tobacco, pipes filled with tobacco, and bidis. Exclusive use of e-cigarettes (*n* = 108), cigar products (*n* = 169), hookah or waterpipe tobacco (*n* = 60), pipes filled with tobacco (*n* = 56), and bidis (*n* = 38) were not examined separately due to the small sample sizes for each group, which is consistent with national tobacco use patterns among U.S. adults in this age group [11].

#### 2.2.2. Sleep Duration

Participants reported sleep duration in response to the question, “On average, how many hours of sleep do you get in a 24 h period?” This question was derived from the CDC’s BRFSS [21] and has been previously validated in prior research [22]. Based on the National Sleep Foundation’s sleep duration recommendations for adults, including older adults [5], responses were categorized into either insufficient sleep duration, defined as less than seven hours of sleep, or sufficient sleep duration, defined as seven hours or more of sleep.

#### 2.2.3. Sleep Problems

Participants reported sleep problems in response to the yes/no question, “Please rate each of the following where “0” indicates that the item is not a problem or concern and “10” indicates it is a major problem or concern.—Your sleep in the past week.” Higher scores indicate more severe sleep problems. Scores ranging from 5 to 10 were defined as experiencing sleep problems. This question was part of a symptom-rating scale that separately rated other health problems, such as pain and shortness of breath [23,24].

#### 2.2.4. Sociodemographic Covariates

Participants’ sociodemographic covariates included age, modeled as a continuous variable; race and ethnicity, categorized as non-Hispanic Black or Hispanic; educational attainment, categorized as high school or less, some college or a 2-year degree, or a 4-year college degree or higher; marital status, categorized as married or partnered, never married, divorced or separated, or widowed; and health insurance coverage, defined as having any commercial or public health insurance, categorized as yes or no. BMI was categorized as normal weight, defined as 18.5–24.9 kg/m^2^; overweight, defined as 25.0–29.9 kg/m^2^; or obese, defined as 30 kg/m^2^ or higher. Participants also reported the number of physician-diagnosed chronic conditions, including asthma, emphysema, or other chronic respiratory conditions, and heart disease [19]. These participant characteristics, such as BMI and number of chronic conditions, were included as covariates because they could potentially be confounders of sleep quantity and quality in the analytic models [25].

### 2.3. Statistical Analysis

Descriptive statistics were calculated to summarize participant characteristics, including current tobacco use status, insufficient sleep duration, sleep problems, and sociodemographic variables. Chi-square and one-way ANOVA tests were initially conducted to assess the associations between sociodemographic covariates and current tobacco use status among participants. Two separate multivariable logistic regression models were fitted to examine the association between current tobacco use status and either insufficient sleep duration or sleep problems as the main dependent variable, while adjusting for sociodemographic covariates, including participants’ age, race and ethnicity, educational attainment, marital status, health insurance coverage, BMI status, and number of chronic conditions. Adjusted odds ratios (AORs) and 95% confidence intervals (95%CI) were computed for each independent variable entered into the models, with a significance level set at *p* < 0.05. All analyses were performed using SPSS version 31.

## 3. Results

### 3.1. Characteristics of Participants Overall and Stratified by Current Tobacco Use Status

Table 1 presents the characteristics of the participants overall and stratified by current tobacco use status. A total of 14.7% of participants reported exclusive cigarette smoking, 7.7% reported dual-tobacco use, and 4.8% reported poly-tobacco use. Participants had an average age of 57.2 years (*SD* = 10.1), and age differed significantly across current tobacco use groups, with those who engaged in poly-tobacco use being the youngest (*M* = 49.5, *SD* = 9.1) and those who did not use tobacco being the oldest (*M* = 58.2, *SD* = 10.2; *p* < 0.001). The majority (58.6%) were of non-Hispanic Black race, and race and ethnicity also varied across groups (*p* < 0.001), with non-Hispanic Black men reporting a greater proportion of exclusive (71.0%) and dual-tobacco use (74.2%), whereas Hispanic men reported a greater proportion of poly-tobacco use (47.5%). Educational attainment varied, with 36.5% obtaining the highest education level of a 4-year college degree or higher and 20.6% obtaining the lowest education level of high school or less. Educational attainment significantly differed by current tobacco use status (*p* < 0.001), with participants with some college or a 2-year degree having the highest proportion of exclusive cigarette smoking (58.0%), participants with the lowest education level having the highest proportion of dual-tobacco use (26.6%), and those with the highest education level having the highest proportion of poly-tobacco use (43.8%).

Over half (52.8%) of the participants were married or partnered, followed by never married (24.5%), and marital status significantly differed by current tobacco use status (*p* < 0.001). Participants who had never been married had the highest proportion of poly-tobacco use (32.5%), whereas men who were divorced or separated had the highest proportion of exclusive cigarette smoking (30.2%) and dual-tobacco use (25.8%). A total of 89.3% of participants had health insurance, and 43.3% were obese and 35.3% were overweight. While there was no difference between health insurance coverage and current tobacco use status, there was a difference based on the BMI status. Specifically, participants of normal weight had the highest proportion of exclusive cigarette smoking (30.6%) and poly-tobacco use (37.5%), whereas participants with an overweight status had the highest proportion of dual-tobacco use (37.5%). Participants had an average of 3.9 chronic conditions (*SD* = 2.9), and the average number of conditions differed significantly across the current tobacco use groups, with those who engaged in poly-tobacco use having the highest average (*M* = 5.0, *SD* = 4.7) and those who did not use tobacco having the lowest average (*M* = 3.8, *SD* = 2.8; *p* = 0.001).

### 3.2. Current Tobacco Use Status by Insufficient Sleep Duration

A total of 46.9% (*n* = 784) of the participants reported insufficient sleep duration < 7 h. Multivariable logistic regression model results (pseudo-R^2^ = 0.072), displayed in Table 2, indicated that participants who engaged in exclusive cigarette smoking (AOR = 1.43, 95%CI = 1.07–1.92, *p* = 0.016) and dual-tobacco use (AOR = 1.56, 95%CI = 1.06–2.28, *p* = 0.024) had greater odds of insufficient sleep duration than those who did not use tobacco.

Concerning significant covariates, participants with BMI indicating obesity had significantly higher odds of insufficient sleep duration (AOR = 1.55, 95%CI = 1.19–2.04, *p* = 0.001) than participants with a normal weight BMI. Conversely, age was inversely associated with insufficient sleep duration, with older participants having significantly lower odds of insufficient sleep (AOR = 0.97, 95%CI = 0.96–0.98, *p* < 0.001). Hispanic men had significantly lower odds of insufficient sleep duration than non-Hispanic Black men (AOR = 0.79, 95%CI = 0.65–0.98, *p* = 0.031). Participants with health insurance had lower odds of insufficient sleep duration than participants without health insurance (AOR = 0.55, 95%CI = 0.40–0.77, *p* < 0.001).

### 3.3. Current Tobacco Use Status by Sleep Problems

A total of 46.2% (*n* = 771) of the participants reported sleep problems. Multivariable logistic regression model results (pseudo-R^2^ = 0.107) predicting sleep problems are presented in Table 3.

Participants who engaged in dual-tobacco use (AOR = 1.96, 95%CI = 1.32–2.91, *p* < 0.001) and poly-tobacco use (AOR = 1.66, 95%CI = 1.01–2.74, *p* = 0.045) had significantly higher odds of sleep problems than those who did not use tobacco. Concerning significant covariates, Hispanic participants had higher odds of sleep problems than non-Hispanic Black men (AOR = 1.36, 95%CI = 1.10–1.68, *p* = 0.004). Participants with BMI indicating obesity had significantly increased odds of sleep problems compared to participants with a normal weight BMI (AOR = 1.33, 95%CI = 1.01–1.75, *p* = 0.041). Conversely, participants’ age was associated with lower odds of reporting sleep problems (AOR = 0.96, 95%CI = 0.95–0.97, *p* < 0.001).

## 4. Discussion

The current study found that dual-tobacco and poly-tobacco use were associated with a higher likelihood of sleep problems compared to non-tobacco use among a sample of non-Hispanic Black and Hispanic men with at least one chronic condition. Additionally, exclusive cigarette smoking and dual-tobacco use were both associated with insufficient sleep duration. These results highlight how tobacco use patterns and nicotine intake may be differentially related to distinct sleep health outcomes. Nicotine disrupts sleep through multiple biological and behavioral mechanisms. As a central nervous system stimulant, nicotine increases arousal by activating cholinergic pathways and promoting the release of neurotransmitters that regulate the sleep–wake cycle, which may contribute to poorer sleep duration and quality among current tobacco users compared to non-users [26]. The National Sleep Foundation identifies sleep latency as a key indicator of sleep quality [3]. Consequently, tobacco users may experience nocturnal withdrawal as nicotine levels decline overnight, leading to increased sleep latency, wake time, and nighttime arousals [27]. Respiratory effects further compound these disruptions, as smoking is associated with airway inflammation, reduced lung function, and an increased risk of sleep-disordered breathing [28].

Importantly, current tobacco use patterns are clinically relevant to sleep outcomes. Exclusive users of a single tobacco product may experience more predictable exposure, whereas dual-tobacco and poly-tobacco users often have more frequent tobacco use and higher overall nicotine intake [29]. Although the current study did not measure nicotine exposure, it is possible that nicotine intake differences may contribute to the associations found between current tobacco use patterns and the sleep outcomes. This hypothesis should be considered in future research that uses objective measures of current tobacco use, such as cotinine, the widely used nicotine metabolite [30]. Further, prior research shows that multi-product tobacco use is associated with poorer sleep duration and poorer sleep quality compared to single product use or non-tobacco use [31,32,33]. These findings are particularly relevant for racially and ethnically diverse men with chronic conditions, who may already be at an elevated risk for sleep problems due to comorbidities such as cardiovascular disease, diabetes, and depression [34,35,36]. In this population, the combined effects of nicotine exposure and sleep disruption may exacerbate chronic disease progression and contribute to poorer overall health outcomes [32,37,38]. Future research should consider biochemically validating tobacco use and assessing levels of nicotine metabolites (e.g., cotinine [30]) to further elucidate the associations found in the current study.

Concerning key sociodemographic covariates, there was an inverse association between age and poor sleep outcomes, such that increasing age was associated with lower odds of both insufficient sleep and sleep problems. These findings suggest that older age may be protective within this sample and are consistent with national research indicating that middle-aged adults often experience shorter sleep duration and poorer sleep quality than older adults with and without chronic disease burden (e.g., diabetes, obesity) [39]. Other research reported that middle-aged adults have the shortest mean sleep duration relative to older adults, potentially due to family- and work-related weekly responsibilities [40]. Additionally, racial and ethnic differences in the sleep outcomes were observed. Hispanic men had lower odds of insufficient sleep duration, but higher odds of sleep problems compared with non-Hispanic Black men. This pattern suggests that sleep quantity and quality may be differentially experienced across the two racial and ethnic groups. A prior report indicated that sleep health disparities, including those related to race and ethnicity, are associated with factors such as discrimination, socioeconomic conditions, neighborhood environments, and cultural sleep norms [30].

Obesity was significantly associated with both insufficient sleep and sleep problems. Several biological mechanisms may explain this association. Obesity and smoking are risk factors for obstructive sleep apnea, a condition characterized by repeated airway obstruction during sleep that leads to frequent awakenings and fragmented sleep [41]. In addition, excess adipose tissue contributes to chronic low-grade inflammation through the release of pro-inflammatory cytokines, which may disrupt circadian regulation and impair overall sleep quality [42]. Health insurance coverage was associated with lower odds of insufficient sleep duration but not sleep problems. One possible explanation is that access to healthcare facilitates better management of chronic conditions, which may support adequate sleep duration. Sleep problems are often influenced by factors beyond healthcare access, including psychosocial stress, financial strain, and neighborhood conditions, which may attenuate the protective effects of health insurance coverage [43,44,45,46]. Overall, the current study’s findings underscore the importance of considering tobacco use patterns in addition to sociodemographic and other key health factors when addressing potential sleep health disparities among racially and ethnically diverse men with chronic conditions.

This study has several strengths, including the use of a large sample of non-Hispanic Black and Hispanic men with chronic conditions, who are historically understudied populations, to assess multiple current tobacco use categories moving beyond exclusive cigarette smoking with sleep quantity and sleep quality measures. However, this study also has limitations that should be considered when interpreting its findings. This study employed a cross-sectional, self-reported survey design, which limits causal inference and may introduce recall or reporting biases. Additionally, temporal relationships could not be established, and thus, bidirectional relationships of the sleep outcomes predicting tobacco use patterns are possible [47]. Future research should employ a longitudinal design and include objective sleep measures, such as actigraphy or polysomnography. While we included several important sociodemographic covariates, there may be potential confounders that were not assessed (e.g., work schedules) that may influence current tobacco use and sleep patterns in the study population. Future studies should consider other contextual factors that may influence these associations.

### Study Implications

Several public health and clinical implications of these findings should be considered. First, healthcare professionals caring for racially and ethnically diverse men with chronic conditions should routinely screen for both current tobacco use and poor sleep health patterns, such as insufficient sleep duration. Second, tobacco prevention and cessation interventions may benefit from adding sleep health screening and counseling components, especially for those who engage in dual-tobacco and poly-tobacco use behaviors and thus have increased odds of experiencing poor sleep quantity and sleep quality. Third, community-based organizations, public health organizations, and healthcare systems that offer chronic disease prevention and management programs should consider the suggested integrated approaches that address current tobacco use and sleep health concurrently, instead of as individual public health problems. These comprehensive preventive intervention efforts may help improve chronic disease management and promote better long-term health outcomes among non-Hispanic Black and Hispanic men with chronic conditions.

## 5. Conclusions

The current study assessed the role of exclusive, dual-, and poly-tobacco use in influencing sleep duration and sleep problems among non-Hispanic Black and Hispanic men with chronic conditions. The observed associations indicate that exclusive smoking and dual-tobacco use predict insufficient sleep duration and dual-tobacco and poly-tobacco use predict sleep problems among participants in this sample. Taken together, these findings suggest that cigarette smoking, whether exclusively or in combination with other tobacco products, may exacerbate sleep health disparities and highlight the importance of accounting for complex tobacco use patterns in research and clinical practice. Targeted interventions focusing on the compounded burden of chronic diseases, tobacco use, and poor sleep are encouraged to improve sleep health disparities and long-term outcomes among men with chronic conditions who are from racially and ethnically diverse backgrounds.

## Figures and Tables

**Table 1:** Characteristics of participants overall and stratified by current tobacco use status among non-Hispanic Black men and Hispanic men with chronic conditions.

		Current Tobacco Use Status				
	Overall (*N* = 1670)	Non-Tobacco Use (*n* = 1217)	Exclusive Cigarette Smoking (*n* = 245)	Dual-Tobacco Use (*n* = 128)	Poly-Tobacco Use (*n* = 80)	*p*-Value
Participant Characteristic	*n* (%) ^a^	*n* (%) ^a^	*n* (%) ^a^	*n* (%) ^a^	*n* (%) ^a^	
Age in Years, *M* (*SD*)	57.2 (10.1)	58.2 (10.2)	56.3 (9.2)	53.4 (8.6)	49.5 (9.1)	<0.001
Race and Ethnicity						<0.001
Non-Hispanic Black	979 (58.6)	668 (54.9)	174 (71.0)	95 (74.2)	42 (52.5)	
Hispanic	691 (41.4)	549 (45.1)	71 (29.0)	33 (25.8)	38 (47.5)	
Educational Attainment						<0.001
High school or less	344 (20.6)	235 (19.3)	59 (24.1)	34 (26.6)	16 (20.0)	
Some college or 2-year degree	716 (42.9)	486 (39.9)	142 (58.0)	59 (46.1)	29 (36.3)	
4-year college degree or higher	610 (36.5)	496 (40.8)	44 (18.0)	35 (27.3)	35 (43.8)	
Marital Status						<0.001
Married or partnered	881 (52.8)	708 (58.2)	81 (33.1)	47 (36.7)	45 (56.3)	
Never married	409 (24.5)	271 (22.3)	72 (29.4)	40 (31.3)	26 (32.5)	
Divorce or separated	317 (19.0)	201 (16.5)	74 (30.2)	33 (25.8)	9 (11.3)	
Widowed	63 (3.8)	37 (3.0)	18 (7.3)	8 (6.3)	0 (0.0)	
Health Insurance Coverage						0.074
No	179 (10.7)	120 (9.9)	30 (12.2)	14 (10.9)	15 (18.8)	
Yes	1491 (89.3)	1097 (90.1)	215 (87.8)	114 (89.1)	65 (81.3)	
BMI Status (kg/m^2^)						<0.001
Normal weight (18.5–24.9)	358 (21.4)	226 (18.6)	75 (30.6)	27 (21.1)	30 (37.5)	
Overweight (25.0–29.9)	589 (35.3)	418 (34.3)	93 (38.0)	48 (37.5)	30 (37.5)	
Obese (≥30)	723 (43.3)	573 (47.1)	77 (31.4)	53 (41.4)	20 (25.0)	
Number of Chronic Conditions, *M* (*SD*)	3.9 (2.9)	3.8 (2.8)	4.1 (2.5)	4.0 (3.1)	5.0 (4.7)	0.001

Abbreviations: *M*, mean; *SD*, standard deviation; BMI, body mass index. ^a^ Percent refers to column percent unless otherwise noted.

**Table 2:** Adjusted logistic regression model results for current tobacco use status by insufficient sleep duration among non-Hispanic Black men and Hispanic men with chronic conditions.

	Insufficient Sleep Duration of <7 h ^a^		
Participant Characteristic	*n* (%) ^b^	AOR (95%CI)	*p*-Value
Current Tobacco Use Status			
Non-tobacco use	531 (43.6)	Ref	
Exclusive cigarette smoking	135 (55.1)	1.43 (1.07–1.92)	0.016
Dual-tobacco use	76 (59.4)	1.56 (1.06–2.28)	0.024
Poly-tobacco use	42 (52.5)	1.23 (0.76–1.99)	0.394
Age in Years, *M* (*SD*)	55.4 (9.3)	0.97 (0.96–0.98)	<0.001
Race and Ethnicity			
Non-Hispanic Black	487 (49.7)	Ref	
Hispanic	297 (43.0)	0.79 (0.65–0.98)	0.031
Educational Attainment			
High school or less	172 (50.0)	Ref	
Some college or 2-year degree	353 (49.3)	1.01 (0.77–1.32)	0.945
4-year college degree or higher	259 (42.5)	0.89 (0.67–1.17)	0.393
Marital Status			
Married or partnered	379 (43.0)	Ref	
Never married	207 (50.6)	0.99 (0.77–1.28)	0.935
Divorced or separated	165 (52.1)	1.26 (0.96–1.65)	0.094
Widowed	33 (52.4)	1.59 (0.94–2.71)	0.086
Health Insurance Coverage			
No	113 (63.1)	Ref	
Yes	671 (45.0)	0.55 (0.40–0.77)	<0.001
BMI Status (kg/m^2^)			
Normal weight (18.5–24.9)	154 (43.0)	Ref	
Overweight (25.0–29.9)	262 (44.5)	1.21 (0.92–1.60)	0.169
Obese (≥30)	368 (50.9)	1.55 (1.19–2.04)	0.001
Number of Chronic Conditions, *M* (*SD*)	3.9 (2.8)	1.00 (0.96–1.03)	0.833

Abbreviations: AOR, adjusted odds ratio; CI, confidence interval; *M*, mean; *SD*, standard deviation; BMI, body mass index. ^a^ The model had pseudo-R^2^ = 0.072, indicating relatively modest model fit. ^b^ Percent refers to row percent unless otherwise noted.

**Table 3:** Adjusted logistic regression model results for current tobacco use status by sleep problems among non-Hispanic Black men and Hispanic men with chronic conditions.

	Sleep Problems ^a^		
Participant Characteristic	*n* (%) ^b^	AOR (95%CI)	*p*-Value
Current Tobacco Use Status			
Non-tobacco use	523 (43.0)	Ref	
Exclusive cigarette smoking	116 (47.3)	1.15 (0.85–1.55)	0.360
Dual-tobacco use	80 (62.5)	1.96 (1.32–2.91)	<0.001
Poly-tobacco use	52 (65.0)	1.66 (1.01–2.74)	0.045
Age in Years, *M* (*SD*)	54.7 (9.5)	0.96 (0.95–0.97)	<0.001
Race and Ethnicity			
Non-Hispanic Black	424 (43.3)	Ref	
Hispanic	347 (50.2)	1.36 (1.10–1.68)	0.004
Educational Attainment			
High school or less	168 (48.8)	Ref	
Some college or 2-year degree	335 (46.8)	0.98 (0.75–1.29)	0.890
4-year college degree or higher	268 (43.9)	0.92 (0.69–1.22)	0.565
Marital Status			
Married or partnered	397 (45.1)	Ref	
Never married	199 (48.7)	0.87 (0.67–1.13)	0.299
Divorce or separated	147 (46.4)	1.00 (0.76–1.32)	0.982
Widowed	28 (44.4)	1.20 (0.70–2.06)	0.514
Health Insurance Coverage			
No	102 (57.0)	Ref	
Yes	669 (44.9)	0.77 (0.55–1.07)	0.121
BMI Status (kg/m^2^)			
Normal weight (18.5–24.9)	149 (41.6)	Ref	
Overweight (25.0–29.9)	268 (45.5)	1.29 (0.97–1.70)	0.079
Obese (≥30)	354 (49.0)	1.33 (1.01–1.75)	0.041
Number of Chronic Conditions, *M* (*SD*)	4.3 (3.0)	1.09 (1.05–1.13)	<0.001

Abbreviations: AOR, adjusted odds ratio; CI, confidence interval; BMI, body mass index. ^a^ The model had pseudo-R^2^ = 0.107, indicating modest model fit. ^b^ Percent refers to row percent unless otherwise noted.

## Data Availability

The data supporting the findings of this study are subject to restrictions. These data were obtained through the State of Texas and may be made available by the authors upon reasonable request and with permission from the State of Texas.

## Abbreviations

The following abbreviations are used in this manuscript:

AOR	Adjusted odds ratio
CI	Confidence interval
BMI	Body mass index
M	Mean
SD	Standard deviation
